# A Tripartite, Hierarchical Sigma Factor Cascade Promotes Hormogonium Development in the Filamentous Cyanobacterium Nostoc punctiforme

**DOI:** 10.1128/mSphere.00231-19

**Published:** 2019-05-01

**Authors:** Alfonso Gonzalez, Kelsey W. Riley, Thomas V. Harwood, Esthefani G. Zuniga, Douglas D. Risser

**Affiliations:** a Department of Biology, University of the Pacific, Stockton, California, USA; University of Iowa

**Keywords:** Nostoc punctiforme, development, gliding motility, hormogonia, sigma factors, type IV pili

## Abstract

Cyanobacteria are prokaryotes capable of oxygenic photosynthesis, and frequently, nitrogen fixation as well. As a result, they contribute substantially to global primary production and nitrogen cycles. Furthermore, the multicellular filamentous cyanobacteria in taxonomic subsections IV and V are developmentally complex, exhibiting an array of differentiated cell types and filaments, including motile hormogonia, making them valuable model organisms for studying development. To investigate the role of sigma factors in the gene regulatory network (GRN) controlling hormogonium development, a combination of genetic, immunological, and time-resolved transcriptomic analyses were conducted in the model filamentous cyanobacterium Nostoc punctiforme, which, unlike other common model cyanobacteria, retains the developmental complexity of field isolates. The results support a model where the hormogonium GRN is driven by a hierarchal sigma factor cascade, with *sigJ* activating the expression of both *sigC* and *sigF,* as well as a substantial portion of additional hormogonium-specific genes, including those driving changes to cellular architecture. In turn, *sigC* regulates smaller subsets of genes for several processes, plays a dominant role in promoting reductive cell division, and may also both positively and negatively regulate *sigJ* to reinforce the developmental program and coordinate the timing of gene expression, respectively. In contrast, the *sigF* regulon is extremely limited. Among genes with characterized roles in hormogonium development, only *pilA* shows stringent *sigF* dependence. For *sigJ*-dependent genes, a putative consensus promoter was also identified, consisting primarily of a highly conserved extended −10 region, here designated a J-Box, which is widely distributed among diverse members of the cyanobacterial lineage.

**IMPORTANCE** Cyanobacteria are integral to global carbon and nitrogen cycles, and their metabolic capacity coupled with their ease of genetic manipulation make them attractive platforms for applications such as biomaterial and biofertilizer production. Achieving these goals will likely require a detailed understanding and precise rewiring of these organisms’ GRNs. The complex phenotypic plasticity of filamentous cyanobacteria has also made them valuable models of prokaryotic development. However, current research has been limited by focusing primarily on a handful of model strains which fail to reflect the phenotypes of field counterparts, potentially limiting biotechnological advances and a more comprehensive understanding of developmental complexity. Here, using Nostoc punctiforme, a model filamentous cyanobacterium that retains the developmental range of wild isolates, we define previously unknown definitive roles for a trio of sigma factors during hormogonium development. These findings substantially advance our understanding of cyanobacterial development and gene regulation and could be leveraged for future applications.

## Abstract (precis)

Cyanobacteria are integral to global carbon and nitrogen cycles, and their metabolic capacity coupled with their ease of genetic manipulation make them attractive platforms for applications such as biomaterial and biofertilizer production. Achieving these goals will likely require a detailed understanding and precise rewiring of these organisms’ GRNs. The complex phenotypic plasticity of filamentous cyanobacteria has also made them valuable models of prokaryotic development. However, current research has been limited by focusing primarily on a handful of model strains which fail to reflect the phenotypes of field counterparts, potentially limiting biotechnological advances and a more comprehensive understanding of developmental complexity. Here, using Nostoc punctiforme, a model filamentous cyanobacterium that retains the developmental range of wild isolates, we define previously unknown definitive roles for a trio of sigma factors during hormogonium development. These findings substantially advance our understanding of cyanobacterial development and gene regulation and could be leveraged for future applications.

## INTRODUCTION

Cyanobacteria are prokaryotes capable of oxygenic photosynthesis and as a result are integral to global primary production. Because many species are able to fix nitrogen, they also contribute substantially to global nitrogen cycles, especially in symbiotic associations with eukaryotic partners (1). Furthermore, the multicellular filamentous cyanobacteria in taxonomic subsections IV and V can develop an array of differentiated cell types and filaments, including nitrogen-fixing heterocysts, spore-like akinetes, and motile hormogonia, making them valuable model organisms for studying development (2). Both hormogonia and heterocysts play essential roles in the establishment of nitrogen-fixing symbioses (3–6). The photosynthetic and nitrogen-fixing capacity of cyanobacteria coupled with their relative ease of genetic manipulation make them attractive platforms for applications such as production of biofuel and biomaterial, as well as biofertilizer, including through the possible engineering of artificial nitrogen-fixing symbioses with crop plants (7). Achieving these goals will likely require a detailed understanding and precise rewiring of the gene regulatory networks (GRNs) that control these organisms.

The association of either a housekeeping sigma factor or one of several alternative sigma factors with a core RNA polymerase to initiate the transcription of distinct gene sets is perhaps the most fundamental level of gene regulation in bacteria (8). In cyanobacteria, the role of alternative sigma factors has been studied extensively, primarily in a handful of unicellular model organisms and in the filamentous model cyanobacterium Nostoc (Anabaena) sp. strain PCC 7120 (here, Nostoc 7120) (9). While too extensive to cover in detail here, studies on unicellular cyanobacteria have identified roles for several alternative sigma factors, but of particular relevance to this study, there appears to be substantial functional redundancy among the group 2 sigma factors (10), and the group 3 sigma factor SigF is essential for motility (11, 12). In the filamentous cyanobacterium *Nostoc* 7120, the group 2 sigma factors SigC and SigE have been implicated in, but are individually dispensable for, heterocyst development (13, 14), while the group 3 sigma factor SigJ has been associated with exopolysaccharide (EPS) production, desiccation tolerance, and photoprotection (15, 16). More recently, a study using Nostoc punctiforme strain ATCC 29133 (=PCC 73102) provided substantial evidence that the group 4 sigma factor SigG is involved in cell envelope repair (17).

However, because *Nostoc* 7120 is incapable of differentiating hormogonia or akinetes, studies with this model organism are limited in their ability to inform our understanding of the role alternative sigma factors play in the complex development of filamentous cyanobacteria. Because N. punctiforme is genetically tractable but still displays the full range of developmental diversity possessed by counterparts in the field, it can be employed to overcome this limitation. Here, using *N. punctiforme*, we provide evidence that the hormogonium GRN in filamentous cyanobacteria involves the hierarchical transcriptional activation of *sigC* and *sigF* by *sigJ*, with each sigma factor playing a distinct and essential role in the developmental program.

## RESULTS

### *sigC, sigF,* and *sigJ* are essential for hormogonium development and motility.

**FIG 1:**
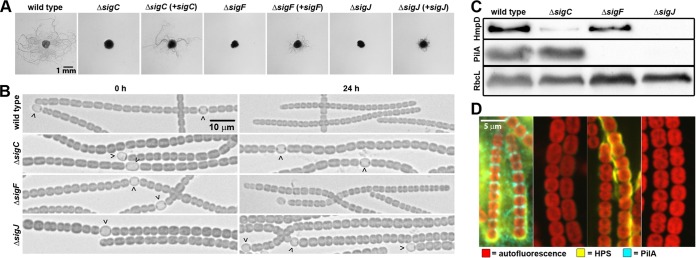
Phenotypic analysis of sigma factor deletion strains. (A) Plate motility assays of the wild type, deletion strains, and complemented deletion strains with deleted genes expressed in *trans* from their native promoters on a replicative shuttle vector (as indicated). (B) Light micrographs of the filament morphology for the wild type and deletion strains (as indicated) at 0 h and 24 h post-hormogonium induction (as indicated). Carets indicate the presence of heterocysts attached to filaments. (C) Immunoblot analysis of HmpD, PilA, and RbcL in the wild-type and deletion strains (as indicated, strain labels correspond to both panels C and D) 24 h after hormogonium induction. RbcL is the large subunit of RuBisCO and serves as a protein loading control. (D) Immunofluorescence and lectin staining analysis of extracellular PilA and HPS. Depicted are merged images of fluorescence micrographs acquired using a 63× objective lens from cellular autofluorescence (red), PilA immunofluorescence (cyan), and UEA-fluorescein-stained HPS (yellow) for various strains (as indicated) 24 h after hormogonium induction.

To date, several genes have been characterized in the literature as being required for normal hormogonium development and motility in *N. punctiforme*, including genes encoding components of the type IV pilus (T4P) motor (*pilA-C*, *pilQ*, *pilT1*, and *pilT2*) (18), proteins involved in the synthesis and secretion of a hormogonium-specific polysaccharide (HPS) (*hpsA-K*) (19), the Hmp chemotaxis-like (*hmpA-F*) (3, 20) and partner-switching (*hmpU-W*) (21) systems, and a putative *O*-linked β-*N*-acetylglucosamine transferase gene (*ogtA*) (22). However, none of these genes are required for the earliest stages of hormogonium development. As part of an ongoing transposon mutagenic screen (22), two nonmotile isolates were identified, TNM14139 and TNM14211, that harbored transposon insertions in *sigC*. Previous transcriptomic studies have also reported enhanced expression of *sigC*, *sigF*, and *sigJ* in developing hormogonia (19, 23, 24). Based on these data, we investigated the role of these sigma factors in hormogonium development by mutational analysis. In-frame deletion of either *sigC*, *sigF*, or *sigJ* completely abolished motility, as assessed by the failure to display colony spreading in plate motility assays and by the absence of motility for individual filaments in time-lapse microscopy assays (Fig. 1A; see also Movie S1 in the supplemental material). Based on genomic context (25) and transcriptomic data (19, 23, 24), *sigC* and *sigJ* appear to be monocistronic, while *sigF* may be cotranscribed with two downstream genes encoding conserved hypothetical proteins. For each deletion strain, reintroduction of the corresponding sigma factor in *trans* on a replicative shuttle vector under the control of its native promoter was sufficient to restore motility, confirming that the deletion of each sigma factor was responsible for the observed phenotype (Fig. 1A). Complementation did not typically restore wild-type levels of motility, a result reported for several other nonmotile *N. punctiforme* mutants complemented in a similar manner (20–22), possibly indicating that precise gene dosage is essential for optimal motility.

10.1128/mSphere.00231-19.2 MOVIE S1 Time-lapse microscopy of the wild-type and sigma factor deletion strains (as indicated). Download Movie S1, AVI file, 7.5 MB. Copyright © 2019 Gonzalez et al. 2019 Gonzalez et al. This content is distributed under the terms of the Creative Commons Attribution 4.0 International license.

To further assess the role of each sigma factor, the filament morphology of each deletion strain was assessed prior to and following induction for hormogonia (Fig. 1B and S1A). There was no obvious growth defect when cells were grown diazotrophically prior to induction, and morphologically distinct heterocysts were present in each strain (Fig. 1B and S1A), indicating that these sigma factors are not required for normal growth, heterocyst development, or nitrogen fixation. The only notable difference prior to induction was a significant increase in cell size (Fig. S1A), which was most pronounced in the Δ*sigC* mutant strain and slightly less so in the Δ*sigJ* mutant, as well as the rare occurrence (less than 1%) of extremely elongated rod-shaped cells in the Δ*sigC* mutant strain (Fig. S1B). Following induction, wild-type *N. punctiforme* differentiates hormogonia, the morphologically distinct markers of which include (i) smaller cell size as a result of reductive cell division, (ii) the loss of heterocysts from the filaments, (iii) shorter filament length due to fragmentation, and (iv) changes in cell morphology, including a transition from more coccoid to rod-shaped cells and the appearance of tapered cells at the filament termini (Fig. 1B and S1A). Although nonmotile, the Δ*sigF* mutant strain differentiated morphologically distinct hormogonia that dismembered their heterocysts and underwent reductive cell division, producing smaller cells characteristic of hormogonia (Fig. 1B and S1A). In contrast, the Δ*sigC* and Δ*sigJ* mutant strains failed to display any of the morphological markers of hormogonia following induction, including a lack of reduction in cell size or the loss of heterocysts (Fig. 1B and S1A). One additional observation of note was the tendency of the Δ*sigJ* mutant strain to aggregate following hormogonium induction despite the lack of obvious morphological changes (Movie S1). These results indicate that both *sigC* and *sigJ* may be required for an early stage in hormogonium development, while *sigF* is late acting.

10.1128/mSphere.00231-19.3 FIG S1 Analysis of hormogonium morphology. (A) Cell length and % filaments with heterocysts in the wild type and each deletion strain (*n* = 3; error bars, standard deviation [SD]). *, *P* < 0.05; **, *P* < 0.01 as determined by two-tailed Student’s *t*-test between the wild type and each deletion strain at the corresponding time point. (B) Appearance of abnormal cell morphology in the Δ*sigC* mutant strain. Caret indicates an example of an abnormally elongated rod-shaped cell. Download FIG S1, PDF file, 1.2 MB. Copyright © 2019 Gonzalez et al. 2019 Gonzalez et al. This content is distributed under the terms of the Creative Commons Attribution 4.0 International license.

To further define the role of each sigma factor in the hormogonium GRN, immunoblotting was used to analyze the expression of the hormogonium-specific proteins PilA (major pilin of the T4P) and HmpD (methyl-accepting chemotaxis protein), as well as immunofluorescence, lectin staining, and lectin blotting to analyze the accumulation of extracellular PilA and HPS in each mutant (Fig. 1C and D). In the wild type, PilA and HmpD are specifically expressed in hormogonia (Fig. 1C), and extracellular PilA accumulates at the junctions between the cells, while extracellular HPS accumulates loosely around the filaments (cell-associated) or dissolves into the medium (soluble) (Fig. 1D and S2). The deletion of *sigJ* had the most marked effect on the expression of these hormogonium-specific markers, as it failed to accumulate any detectable levels of cellular PilA and HmpD (Fig. 1C) or extracellular PilA and HPS (Fig. 1D and S2). In contrast, the Δ*sigC* mutant strain accumulated substantially reduced levels of HmpD and wild-type levels of cellular PilA (Fig. 1C) yet failed to display any detectable extracellular PilA or HPS (Fig. 1D and S2). The Δ*sigF* mutant strain accumulated wild-type levels of cellular HmpD but no detectable cellular or extracellular PilA (Fig. 1C) and showed a moderate reduction in the accumulation of extracellular HPS, most of which was more tightly associated with the filaments compared to the wild-type strain (Fig. 1D and S2). Collectively, these results imply that *sigJ* may play an essential role early in the hormogonium GRN and may subsequently promote the expression of *sigF* and *sigC*, which in turn control distinct regulons.

10.1128/mSphere.00231-19.4 FIG S2 Quantification of cell-associated and soluble extracellular HPS (*n* = 3; error bars, SD) in the wild type and each deletion strain 24 h after hormogonium induction. *, *P* < 0.05; **, *P* < 0.01 as determined by two-tailed Student’s *t*-test between the wild type and each deletion strain. Download FIG S2, PDF file, 0.2 MB. Copyright © 2019 Gonzalez et al. 2019 Gonzalez et al. This content is distributed under the terms of the Creative Commons Attribution 4.0 International license.

**FIG 2:**
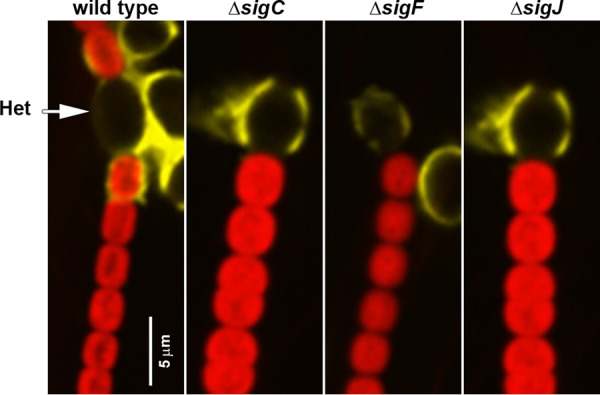
UEA-fluorescein staining of HEP. Depicted are merged images of fluorescence micrographs acquired using a 63× objective lens from cellular autofluorescence (red) and UEA-fluorescein-stained HEP (yellow) for various strains (as indicated) prior to hormogonium induction. The position of heterocysts, which lack autofluorescence, is indicated with an arrow.

The lectin Ulex europaeus agglutinin I (UEA), employed here to detect HPS, was recently demonstrated to bind to the heterocyst envelope as well (20). This staining was shown to be dependent on a functional *hpsE-G* locus, possibly indicating some overlap in the components of the systems involved in the synthesis of heterocyst envelope polysaccharide (HEP) and HPS. However, despite the absence or reduction of accumulated HPS in hormogonium-induced cultures, UEA staining of the heterocyst envelope within vegetative filaments was not disrupted in any of the deletion strains (Fig. 2). Thus, the expression of *hpsE-G* is unlikely to be under stringent regulation by any one of the three sigma factors.

### Defining the regulons of *sigJ, sigC,* and *sigF* by RNA-seq analysis.

**FIG 3:**
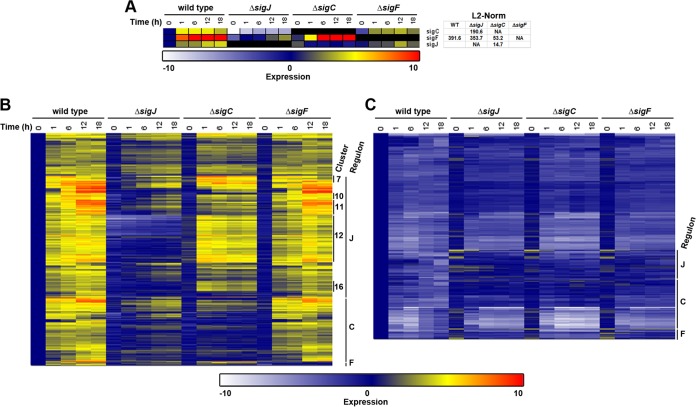
RNA-seq analysis of sigma factor deletion strains. (A) Heat map depicting the expression of each sigma factor over a five-point time course of hormogonium development in both the wild type (WT) and each deletion strain. In all panels, expression = log_2_(average normalized expression for each strain and time point/average normalized expression of the wild type at 0 h). L2-norm values are indicated for each gene and strain for those genes with statistically significant changes in expression in the wild-type time course, or in the sigma factor deletion strain in comparison to the wild type, as determined by BATS. (B and C) Global analysis of upregulated (B) and downregulated (C) genes (as indicated) differentially expressed in developing hormogonia. Assigned sigma factor regulons and notable clusters are indicated.

To further define the relationships between the trio of sigma factors, as well as the extended regulon of each in the hormogonium GRN, RNA sequencing (RNA-seq) analysis and reverse transcription-quantitative PCR (RT-qPCR) were employed to analyze the transcriptome of developing hormogonia over a five-point time course in the wild type and each deletion strain (Fig. 3 and S3 and Data Set S1). Consistent with previous reports (19, 23, 24), the expression of each sigma factor was upregulated in developing hormogonia (Fig. 3A and S3). The deletion of *sigJ* abolished transcriptional activation of both *sigC* and *sigF*. The deletion of *sigC* had relatively little effect on the enhanced expression of *sigF* but prevented the upregulation of *sigJ*. The fact that upregulation of *sigJ* is dependent on *sigC*, and vice versa, implies that *sigJ* and *sigC* may form a positive feedback loop. However, the observation that the transcription of *sigF*, which is also *sigJ* dependent, is largely unaffected in the Δ*sigC* mutant strain, where *sigJ* expression is static, could indicate that posttranscriptional regulation of *sigJ* plays an important role in the activation of *sigJ*-dependent genes. In contrast, the deletion of *sigF* did not substantially alter the expression pattern of either *sigJ* or *sigC*. These results are consistent with a model where the hormogonium GRN involves the hierarchical activation of *sigC* and *sigF* by *sigJ*.

10.1128/mSphere.00231-19.5 FIG S3 RT-qPCR verification of RNA-seq analysis for select genes. Heat map depicting the expression of several hormogonium-specific genes, as determined by either RT-qPCR or RNA-seq (as indicated) over a five-point time course of hormogonium development in both the wild type and each deletion strain. Expression = log_2_(average normalized expression for each strain and time point/average normalized expression of the wild type at 0 h). Genes are arranged based on a combination of hierarchical cluster analysis and manual curation. Download FIG S3, PDF file, 1.2 MB. Copyright © 2019 Gonzalez et al. 2019 Gonzalez et al. This content is distributed under the terms of the Creative Commons Attribution 4.0 International license.

10.1128/mSphere.00231-19.6 DATA SET S1 RNA-seq analysis. (A) Normalized expression data output from Rockhopper. (B) Log_2_-transformed expression values (normalized expression of experimental condition/normalized expression of wild-type strain at T = 0 h [mean of 3 biological replicates]) and statistical output from BATS analysis. (C and D) Upregulated (C) and downregulated (D) genes in the wild-type time course as determined by BATS, with assigned regulons, hierarchical clustering (HCL) order, and assigned cluster numbers. Download Data Set S1, XLSX file, 2.4 MB. Copyright © 2019 Gonzalez et al. 2019 Gonzalez et al. This content is distributed under the terms of the Creative Commons Attribution 4.0 International license.

To define the regulon for each sigma factor in the hormogonium GRN, differentially expressed protein-coding genes were first identified in the wild-type time course of hormogonium development. Subsequently, subsets of these genes with different expression patterns in the sigma factor deletion strains were defined. A total of 601 genes were upregulated and 533 downregulated. This gene set was divided into 4 discrete categories. The first category included genes with expression patterns that were not statistically different in any of the deletion strains from the wild type. Those genes that were differentially expressed in one or more deletion strains were then assigned to a regulon for one of the three sigma factors based on which strain produced the greatest change in expression compared to the wild type, as determined by ranking the L2-norm (i.e., Euclidean norm) values for each gene between the three deletion strains. Finally, a hierarchical cluster analysis was performed to group genes within each category based on similar expression patterns (Fig. 3B and C and Data Set S1C and D). A total of 408 genes were expressed in a similar manner in all strains, with the majority of these downregulated (301 downregulated/107 upregulated). *sigJ* had by far the largest regulon, at 389 genes, the majority of which were normally upregulated in wild-type hormogonia (72 downregulated/317 upregulated), followed by *sigC*, with a total of 300 genes (133 downregulated/167 upregulated), and, finally, *sigF*, with only 37 genes (27 downregulated/10 upregulated). Genes that exhibited *sigC*- or *sigF*-dependent upregulation required *sigJ* for enhanced transcription as well, consistent with the hierarchical activation model proposed above. Notably, the deletion of *sigC* did not markedly disrupt the enhanced expression of most *sigJ*-dependent genes despite the fact that expression of *sigJ* remains static in the Δ*sigC* genetic background. This observation lends additional support to the hypothesis that *sigJ* is regulated posttranscriptionally upon hormogonium induction. However, within the *sigJ* regulon, several clusters displayed a temporal shift in expression in the Δ*sigC* mutant strain, most notably clusters 7, 12, and 16, with transcripts accumulating more rapidly upon induction (Fig. 3B). This may indicate that a component of the *sigC* regulon inhibits the activity of SigJ, possibly serving as a checkpoint to coordinate the timing of expression between *sigJ*- and *sigC*-dependent genes.

**FIG 4:**
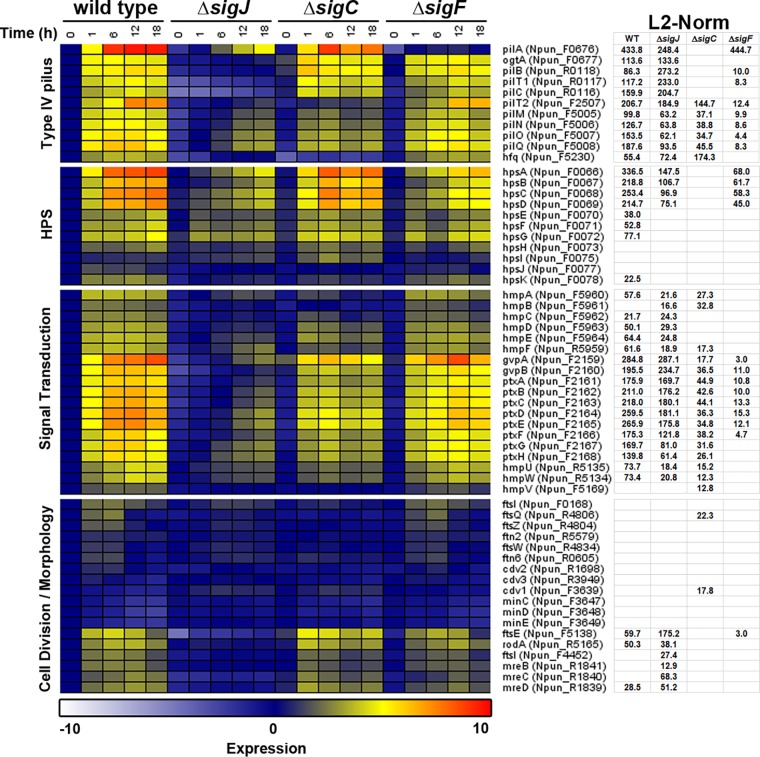
RNA-seq analysis of selected gene sets with previously reported functional implications in hormogonium development. Heat map depicting the expression of each gene over a five-point time course of hormogonium development in both the wild type and each deletion strain. Expression = log_2_(average normalized expression for each strain and time point/average normalized expression of the wild type at 0 h). L2-norm values are indicated for each gene and strain for those genes with statistically significant changes in expression in the wild-type time course, or in the sigma factor deletion strain in comparison to the wild type, as determined by BATS. Genes are arranged based on a combination of hierarchical cluster analysis and manual curation.

In addition to this global analysis, a “biology-guided” approach was taken by analyzing the expression pattern of specific gene sets with characterized roles in hormogonium development or associated cellular processes, including T4P assembly, HPS synthesis and secretion, signal transduction, and cell division and morphology (Fig. 4). The expression patterns for several of these genes were also verified independently by RT-qPCR (Fig. S3). The majority of genes encoding T4P components were most stringently dependent on *sigJ*, including those in the *pilB-C* operon, the *pilM-Q* operon, *pilT2*, and *ogtA*. In contrast, expression of *hfq*, encoding a putative RNA chaperone shown to interact with, and be essential for, T4P function in the unicellular cyanobacterium Synechocystis sp. strain PCC 6803 (26, 27), is most stringently dependent on *sigC*, while expression of the major-pilin-encoding gene *pilA* was strictly *sigF* dependent, consistent with reports from a unicellular cyanobacterium (11, 12). These observations are in accordance with the results from the immunological examination of the T4P system described above, and it may be that in the Δ*sigC* mutant strain, the lack of *hfq* expression accounts for the absence of extracellular PilA despite the relatively robust expression of most other T4P genes and the intracellular accumulation of PilA protein.

Among the genes encoding proteins involved in HPS synthesis and secretion, the expression of *hpsA-D*, encoding a hypothetical membrane protein and pseudopilins, respectively, which may interact with the T4P system to facilitate HPS export, were dependent on both *sigJ* and, to a lesser extent, *sigF*. In contrast, the expression of the downstream genes *hpsE-G*, which encode glycosyltransferases thought to be directly involved in the synthesis of HPS, was only moderately affected by deletion of any one sigma factor, which is consistent with UEA-specific lectin staining of the heterocyst envelope in each of the sigma factor deletion strains, as reported above.

Within the 3 signal transduction systems analyzed, the expression of both the chemotaxis-like *hmp* and *ptx* systems, as well as the *hmp* partner-switching system, is primarily dependent on *sigJ*, consistent with the results from the Western blot analysis of HmpD. This data set also included the *gvpA* and *B* genes, encoding gas vesicle proteins, which are upstream of and cotranscribed with the *ptx* locus, likely as a single operon (28). One notable exception was *hmpV*, encoding a sulfate transporter and anti-sigma factor antagonist (STAS) domain protein that functions as the output of the Hmp partner-switching system, the transcription of which was stringently dependent on *sigC*.

While genes in the T4P, HPS, and signal transduction systems investigated all displayed a similar trend, where transcription was primarily *sigJ* dependent and only a small but critical subset more specifically require *sigC* or *sigF*, this situation was reversed among the gene set controlling cell division. Many of these genes, including one of two *ftsI* homologs (Npun_F0168), *ftsQ*, *ftsZ*, and *ftn6*, show a transient increase in expression, primarily between 1 and 6 hours postinduction, that was completely abolished in the Δ*sigC* mutant strain and disrupted to a lesser extent in the absence of *sigJ*. In contrast, the expression of *ftsE* in developing hormogonia was more sustained and only required *sigJ* for enhanced transcription. While the differential expression for most of these genes was not identified as statistically significant based on the analysis applied here, it is highly consistent with previously published microarray-based studies (19, 23, 24), as well as the RT-qPCR analysis performed for selected genes from this group (Fig. S3); it is also consistent with the morphological observation mentioned above that the deletion of *sigC* and, to a lesser extent, s*igJ*, results in increased cell size. As with most of the other processes investigated, genes involved in regulating rod-shaped cell morphology, such as *rodA* and *mreB-D,* were upregulated in hormogonia in a stringently *sigJ*-dependent manner. A second copy of *ftsI* (Npun_F4452) was also found to be regulated specifically by *sigJ*, possibly indicating that the encoded protein is involved in cell morphology rather than cell division.

### Identification of a consensus *sigJ* promoter sequence.

To identify conserved motifs within the promoter regions of hormogonium-specific genes, we manually curated the data following hierarchical cluster analysis and assigned a number to each cluster with 5 or more genes showing a similar transcription profile distinct from their neighbors. Subsequently, genes that were neither monocistronic nor the first member of a polycistronic transcript, as determined by manual examination of the read map data, were removed from the data set, and the promoter regions for the remaining genes (−60 to +20 of the putative transcriptional start site [TSS]) were retrieved and scanned for conserved motifs by MEME (29) (Data Set S2A and B).

10.1128/mSphere.00231-19.7 DATA SET S2 Motif analysis. (A and B) Promoter regions (−60 to +20 of the predicted TSS) for genes upregulated (A) and downregulated (B) in the wild-type time course and motifs identified for each cluster by MEME analysis. Within each promoter, conserved motifs are in boldface and colored blue. (C) Evolutionary conservation of J-Boxes in the promoter regions of *pilB* in cyanobacteria. J-Boxes are in boldface and colored red and were identified based on the occurrence of three consecutive Gs, followed by the highly conserved +2A and +7T with respect to the first G, each of which is underlined. Download Data Set S2, XLSX file, 0.08 MB. Copyright © 2019 Gonzalez et al. 2019 Gonzalez et al. This content is distributed under the terms of the Creative Commons Attribution 4.0 International license.

**FIG 5:**
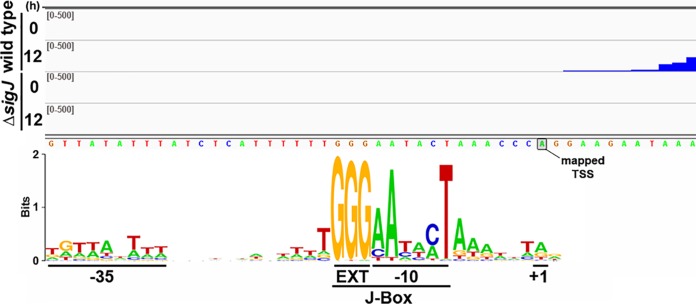
Identification of a consensus *sigJ*-dependent promoter. Read maps depict read coverage in the promoter region of the *gvpA* gene at 0 and 12 h postinduction (as indicated) for both the wild type and Δ*sigJ* mutant strain (as indicated). The TSS as previously defined by 5′RACE (28) is indicated. The consensus *sigJ*-dependent promoter region, as determined by alignment of 93 promoters containing a J-Box, is depicted below. Putative +1, −10, EXT, and −35 regions are indicated.

Most notably, three separate clusters, 7, 12, and 16, within the *sigJ* regulon contained a conserved motif which included some variation on the sequence GGGaAtacT, here designated a J-Box. As previously noted above, each cluster displayed a similar transcription profile across the four strains analyzed, with enhanced transcription in wild-type hormogonia that was almost completely abolished in the Δ*sigJ* mutant strain and temporally shifted to more rapid transcript accumulation in the Δ*sigC* mutant strain (Fig. 3B). The only apparent difference between each cluster was the amplitude of the expression profiles. Of the 118 promoter regions from these three clusters, 93 contained an identifiable motif in the forward orientation with respect to the corresponding gene, and the location of the motif was often in close proximity to the putative −10 region based on the annotated TSSs (Data Set S2A). The conserved trio of Gs within the motifs of these 93 promoters was used to anchor a sequence alignment, revealing what appears to be a consensus SigJ promoter region (Fig. 5).

Because the RNA-seq analysis employed here did not rely on the enrichment of triphosphorylated 5′ transcripts (differential RNA-seq [difSeq]), the predicted transcriptional start sites are likely to be inexact, thus making it difficult to precisely place this consensus sequence with respect to the bona fide TSS for each promoter. However, for *gvpA*, which was a member of this data set, the TSS has previously been mapped by 5′ rapid amplification of cDNA ends (RACE) (28), providing an additional data point to help position this sequence. Read coverage in the *gvpA* promoter region corresponds closely with the previously determined TSS, which is precisely 10 bp upstream of the center of the aAtacT consensus sequence within the J-Box (Fig. 5), indicating that this represents a bona fide −10 promoter region. It is likely that the almost absolutely conserved A and T in this region are the nucleotides flipped out by subdomains 2.3 and 2.4 of SigJ to initiate unwinding of the double-stranded DNA (30) while the absolutely conserved trio of Gs upstream functions as an extended −10 region that interacts with domain 3 of SigJ (31). Unlike the −10 region, the putative −35 region is primarily AT rich and only weakly conserved. This is consistent with previous reports indicating that the −35 region is largely dispensable for promoters containing strong consensus extended −10 regions (32). The promoter of *sigC* was not included in this analysis, but a J-Box was subsequently identified by manual annotation, consistent with direct regulation of *sigC* by SigJ. In contrast, no obvious J-Box was identified in the *sigF* promoter region.

A second motif of note was also identified in a subset of genes from clusters 10 and 11 (Data Set S2A). These genes are dramatically upregulated in hormogonia and appear to be somewhat codependent on both *sigJ* and *sigC* for expression (Fig. 3B). From the available data, it was not apparent whether this motif might represent some portion of the −10/−35 region for these promoters, or perhaps some other regulatory element. No conserved motifs were identified among the downregulated gene set (Data Set S2B).

## DISCUSSION

**FIG 6:**
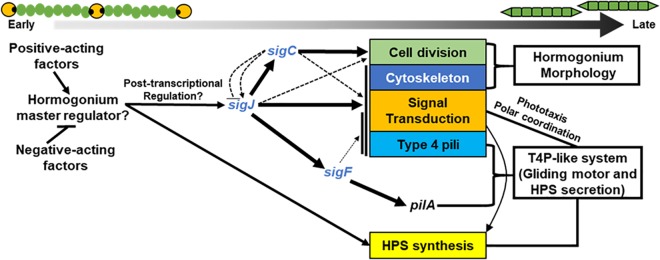
A model depicting the major findings of this report on the role of *sigJ*, *sigC*, and *sigF* in the hormogonium GRN. Arrows indicate positive regulation, and lines with bars indicate negative regulation. The thickness of the arrows represents a combination of the number of genes regulated and the stringency of the regulation.

The concurrence of both the transcriptomic and phenotypic data reported here provides substantial support for a model where hormogonium development is driven by a hierarchal sigma factor cascade (Fig. 6), with *sigJ* activating expression of both *sigC* and *sigF* as well as a substantial portion of additional hormogonium-specific genes, including those driving changes to cellular architecture. In turn, *sigC* specifically regulates smaller subsets of genes for several processes, plays a dominant role in promoting reductive cell division, and may also both positively and negatively regulate *sigJ* to reinforce the developmental program and coordinate the timing of expression during development, respectively. Both *sigJ* and *sigC* also regulate many genes with currently uncharacterized roles in hormogonium development. In contrast, the *sigF* regulon is extremely limited. Among genes with characterized roles in hormogonia, only *pilA* shows stringent *sigF* dependence. The transcript level of *pilA* in hormogonia is extremely abundant, with normalized expression values indicating that it is the second most highly expressed protein-coding gene in the entire genome (12 to 18 h postinduction; Data Set S1A). We speculate that the requirement for extremely high levels of PilA may have necessitated the evolution of this nearly 1:1 ratio between a sigma factor and its target regulon. The genes most directly involved in the synthesis of HPS appear to lie outside the sigma factor cascade, but their expression is likely reinforced by the activity of the Hmp signal transduction systems (19, 21).

The fact that enhanced expression of *sigJ* is dispensable for activation of its downstream targets in the Δ*sigC* genetic background is consistent with a model where posttranscriptional regulation of *sigJ* is a critical early step in activating the hormogonium GRN. While the currently available data cannot differentiate between a posttranscriptional and posttranslational mechanism, posttranslational regulation of sigma factors, often by anti-sigma factors and/or regulated proteolysis, is common (33). A recent effort at heterologous expression of the full complement of *Nostoc* 7120 sigma factors in Escherichia coli was unsuccessful in the case of SigJ (34), possibly indicating inherent instability in the absence of some additional factor(s). This lends support to the theory that SigJ may be subject to regulated proteolysis. It is also notable that an Rsb-like partner-switching system (HmpU-W) was recently implicated in hormogonium development (21). Canonical Rsb-like systems regulate sigma factors via sequestration by an anti-sigma factor (35). Although the Hmp partner-switching system appears to function by an alternative mechanism where the sulfate transporter and anti-sigma factor antagonist (STAS) domain protein HmpV functions as the output to regulate a currently undefined downstream effector, it is conceivable that this system may regulate one or more sigma factors through a nonstandard mechanism. Moving forward, defining the mechanism by which *sigJ* is regulated is an intriguing area for future study.

In the case of *sigJ*, it was also possible to identify a probable consensus promoter sequence and therefore to define those genes that are most likely under direct control of SigJ. Based on these data, SigJ directly regulates ∼100 promoters and ∼200 genes, including *sigC,* but not *sigF*. In contrast, motif searches among promoter regions in the *sigC* and s*igF* regulons failed to yield any identifiable consensus sequences. In the case of *sigF*, this search was hampered by the extremely limited number of genes in the data set. The reason for the failure to identify a consensus sequence among *sigC*-dependent promoters is less clear, but potential contributing factors include (i) the possibility that much of the *sigC*-dependent regulation in hormogonia is indirect, (ii) a higher tolerance for variability in SigC promoters, and (iii) the presence of multiple TSSs for many of the *sigC*-dependent genes, resulting in inaccurate TSS predictions. Moving forward, more precise promoter mapping of individual genes by 5′ RACE/primer extension, or globally by a difSeq approach, has the potential to shed light on the nature of SigF- and SigC-specific promoters.

**FIG 7:**
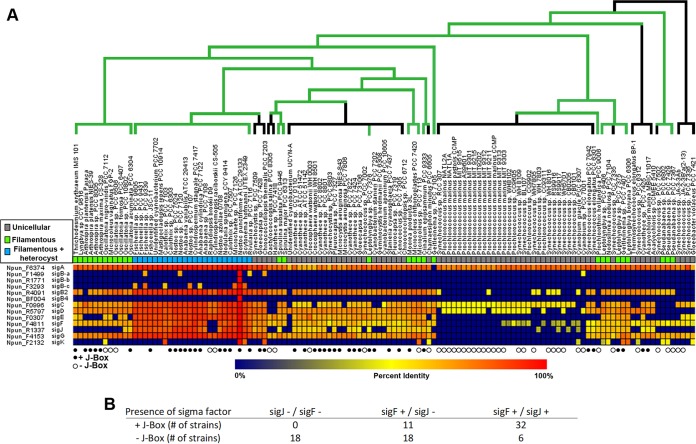
Evolutionary conservation of sigma factors and J-Boxes in cyanobacteria. (A) A heat map depicting the percent identity for orthologs of *N. punctiforme* sigma factors in cyanobacteria, derived from data reported by Cho et al. (20). Species organization and phylogenetic tree based on the phylogeny reported by Shih et al. (51) but depicting the finding, as reported by Schirrmeister et al. (52), that most extant cyanobacteria are derived from a filamentous ancestor. For the phylogenetic tree, green indicates filamentous, and black indicates unicellular. Closed and open circles indicate the presence and absence, respectively, of a J-Box within 100 bp of the *pilB* translational start site for the corresponding strain. Strains without a circle were not included in the analysis. (B) Summary data table on cooccurrence of *sigF* and/or *sigJ* with a J-Box upstream of *pilB*.

It should be noted that the findings from this work differ substantially from previous reports on the role of *sigC* and *sigJ* in filamentous cyanobacteria (13–16). We attribute this primarily to the choice of model organism. As stated in the introduction, *Nostoc* 7120 fails to differentiate hormogonia or akinetes. This is likely due to prolonged culture under laboratory conditions rather than being reflective of the natural state of field isolates, considering that orthologs for all of the hormogonium-related genes currently characterized in the literature are contained in the *Nostoc* 7120 genome (20) and have not been ascribed to an alternative function. Furthermore, microevolutionary loss of motility has been documented for various lab strains of the unicellular cyanobacterium *Synechocystis* sp. strain PCC 6803 (36, 37), and it was recently reported that a field isolate of the nonmotile model unicellular cyanobacterium Synechococcus elongatus PCC 7942 is motile as well (38). A role for *sigJ* in hormogonium development and motility is also more consistent with proposed evolutionary history, given that *sigJ* likely arose via duplication with *sigF*, which is known to regulate motility (9). In fact, given these findings, it is reasonable to speculate that null mutations in *sigC* and/or *sigJ* of *Nostoc* 7120 may underlie the failure to differentiate hormogonia. Thus, the findings of this study provide a compelling example of the power of *N. punctiforme* as a model organism for exploring the biology of filamentous cyanobacteria and more generally highlight the importance of considering how commonly used model organisms reflect their wild counterparts. Given the wide distribution of *sigC*, *sigF*, and *sigJ* orthologs among cyanobacteria (Fig. 7A), these findings may inform our understanding of a wide range of both unicellular and filamentous cyanobacterial species. In fact, J-Boxes could be identified in the promoter region of *pilB* from many cyanobacterial species, primarily those harboring orthologs of *sigJ* (Fig. 7A and B and Data Set S2C), such as the model unicellular cyanobacterium Synechococcus elongatus PCC 7942, but also in several that do not, including Synechocystis sp. strain PCC 6803. For those strains lacking *sigJ*, an ortholog of the evolutionarily related *sigF* was always present (Fig. 7B), implying that in these strains, SigF may be capable of recognizing promoters containing a J-Box. In fact, while not identical, there are apparent similarities between the J-Box and the previously defined SigF-recognized −10 region in *Synechocystis* sp. strain PCC 6803 (11).

The role of *sigC* in hormogonium development also represents a paradigm shift in the current understanding of group 2 sigma factors in cyanobacteria. Previous reports on both unicellular and filamentous cyanobacteria imply substantial functional redundancy among group 2 sigma factors. In the case of filamentous cyanobacteria, this is most evident in the apparently overlapping regulons of *sigC* and *sigE* during heterocyst development (14). This is clearly not the case for the group 2 sigma factor *sigC* during hormogonium development, where stringent promoter recognition of a critical gene set is essential.

Finally, although beyond the focus of this study, the RNA-seq data generated here provide a valuable resource for annotating small regulatory and antisense RNAs that may play a role in the development of *N. punctiforme*, and collectively, the insights on the regulation of cyanobacterial transcription and development can potentially be leveraged to rewire cyanobacterial GRNs for biotechnological applications.

## MATERIALS AND METHODS

### Strains and culture conditions.

For a detailed description of the strains used in this study, refer to Table S1A. *N. punctiforme* ATCC 29133 and its derivatives were cultured in Allan and Arnon medium diluted 4-fold (AA/4), without supplementation of fixed nitrogen, as previously described (24), with the exception that 4 and 10 mM sucralose were added to liquid and solid media, respectively, to inhibit hormogonium formation (39). For small-scale hormogonium induction for phenotypic analysis, the equivalent of 30 μg ml^−1^ chlorophyll *a* (Chl *a*) of cell material from cultures at a Chl *a* concentration of 10 to 20 μg ml^−1^ was harvested at 2,000 × *g* for 3 min, washed two times with AA/4, and resuspended in 2 ml of fresh AA/4 without sucralose. For large-scale hormogonium induction for RNA-seq analysis, this process was repeated but starting with the equivalent of 300 μg ml^−1^ Chl *a* of cell material and resuspension in 50 ml of fresh AA/4. For selective growth, the medium was supplemented with 50 μg ml^−1^ neomycin. Escherichia coli cultures were grown in lysogeny broth (LB) for liquid cultures or LB supplemented with 1.5% (wt/vol) agar for plates. Selective growth medium was supplemented with 50 μg ml^−1^ kanamycin, 50 μg ml^−1^ ampicillin, and 15 μg ml^−1^ chloramphenicol.

10.1128/mSphere.00231-19.1 TABLE S1 Plasmids, strains, and oligonucleotides used in this study. (A) Plasmids and strains used in this study. (B) Oligonucleotides used in this study. Download Table S1, PDF file, 0.3 MB. Copyright © 2019 Gonzalez et al. 2019 Gonzalez et al. This content is distributed under the terms of the Creative Commons Attribution 4.0 International license.

### Plasmid and strain construction.

For a detailed description of the plasmids, strains, and oligonucleotides used in this study, refer to Tables S1A and B. All constructs were sequenced to ensure fidelity.

To construct plasmids for in-frame deletion of *sigC*, *sigF*, and *sigJ*, approximately 900 bp of flanking DNA on either side of the gene and several codons at the beginning and end of each gene were amplified via overlap extension PCR (see Tables S1A and B for details) and cloned into pRL278 (40) as BamHI-SacI fragments using restriction sites introduced on the primers.

To construct mobilizable shuttle vectors containing *sigC, sigF,* or *sigJ* and their respective promoter regions, the coding region and 5′ intergenic region for each gene were amplified via PCR (see Tables S1A and B for details) and subsequently cloned into pAM504 (41) as a BamHI‐SacI fragment using restriction sites introduced on the primers.

Generation of transposon mutants and identification of transposon insertion sites were performed as previously described (22) using plasmid pRL1063a (42). Gene deletions and allelic replacements were performed as previously described (19) with *N. punctiforme* cultures supplemented with 4 mM sucralose to inhibit hormogonium development and enhance conjugation efficiency (22, 39). To construct the Δ*sigC,* Δ*sigJ*, and Δ*sigF* mutant strains, pDDR411, pDDR412, and pKDDR413, respectively, were introduced into wild-type *N. punctiforme,* creating strains UOP131, UOP132, and UOP141, respectively.

### Motility assays.

Both plate and time-lapse motility assays were performed as previously described (18).

### RNA-seq.

Total RNA was extracted from the equivalent of 300 μg ml^−1^ Chl *a* of cell material for each of 3 biological replicates from each strain at time points of 0, 1, 6, 12, and 18 h following hormogonium induction, using previously published methods (24). Subsequent cDNA synthesis and sequencing were performed at the University of California Berkeley QB3 Vincent J. Coates Genomics Sequencing Laboratory using 10 μg of total RNA, as follows. rRNA was depleted using the Ribo-Zero rRNA removal kit (bacteria) (Illumina, Inc.). Directional cDNA libraries were synthesized from the rRNA-depleted samples, sheared to a library size of ∼200 bp, and appended with adapters. All 60 libraries were multiplex sequenced across 6 lanes of an Illumina HiSeq 4000 flow cell, generating 100-bp paired-end reads. Alignment, assembly, normalization, and quantitation of sequencing data were performed using the software package Rockhopper (default parameters) (43), and transcript maps were generated using Integrated Genomics Viewer (44). On average, a total of 24,290,031 reads mapped to the *N. punctiforme* genome (excluding genes coding for rRNA) for each replicate and time point, providing sufficient sequencing depth to detect differential expression of even low-abundance transcripts (45).

Differential expression for each strain, time point, and replicate was calculated as log_2_(normalized expression of experimental condition/normalized expression of wild-type strain at T = 0 h [mean of 3 biological replicates]). Statistical identification of differentially expressed genes in the wild-type time course and between the time course for the wild-type and sigma factor deletion strains was performed using Bayesian Analysis of Time Series (BATS) (default parameters) (46). Differentially expressed genes were subsequently binned into one of four categories. The first contained genes that were differentially expressed during wild-type hormogonium development and whose expression was not statistically different in any of the sigma factor deletion strains. The remaining 3 categories contained genes that were differentially expressed in the wild type but showed an altered expression pattern in one or more of the sigma factor deletion strains. These genes were assigned to one of three bins, one for each sigma factor, based on which deletion strain produced the largest L2-norm value. Each bin was subsequently subjected to a hierarchical cluster analysis (unweighted pair group method using average linkages [UPGMA]) using the software package Genesis (47). Heat maps depicting expression data were generated in Genesis (47). Groups of 5 or more genes with similar expression patterns that were distinct from their neighbors were then manually assigned a cluster number.

### Motif discovery.

For identification of conserved motifs in promoter regions, first, either the wild-type 0-h or 12-h biological replicates were individually analyzed with Rockhopper to predict transcriptional start sites (TSSs) for each gene. The 12-h annotated TSSs were used for analysis of upregulated genes, while the 0-h annotated TSSs were used for downregulated genes, working on the assumption that higher expression levels were more likely to yield accurate TSS determinations. Subsequently, the promoter regions (−60 to +20 of the annotated TSS) were retrieved for genes that were either monocistronic or the first gene of a polycistronic transcript based on visual inspection of the read map data. The promoter regions for each cluster of genes with similar expression profiles (as defined above) were then analyzed by MEME (29) to identify conserved motifs. Only motifs present in the same orientation in at least 50% of the promoters for a given cluster are reported in Data Set S2A and B. Generation of a consensus promoter sequence from promoter regions in clusters 7, 12, and 16, containing a J-Box, was performed using WebLogo (48).

### RT-qPCR.

Five hundred nanograms of total RNA was used to synthesize cDNA with the ProtoScript first-strand cDNA synthesis kit and random hexamer primers (New England BioLabs, Inc.), following the specifications of the manufacturer, after which 1 μl of cDNA was used as the template for qPCR. Transcripts were amplified with the primer sets indicated in Table S1B, using a StepOnePlus real-time PCR system (Applied Biosystems) and SensiFAST SYBR No-ROX kit (Bioline), following the manufacturer’s specifications. Quantification of transcript abundance was calculated from the average of two technical replicates from each of the three biological replicates using the 2^−ΔΔ^*^CT^* method (49), with expression normalized relative to *rnpB*. The primer efficiencies for each primer pair were all greater than 90%.

### Immunoblot and lectin blot analysis.

Preparation of cell material, protein extraction, and detection of PilA, RbcL, and HmpD by immunoblot analysis were performed as previously described (20). Preparation, detection, and quantification of soluble HPS in the culture medium by lectin blotting with biotinylated UEA (Vector Laboratories) were performed as previously described (18).

### Immunofluorescence and fluorescent lectin staining.

Simultaneous detection of PilA and HPS by immunofluorescence and fluorescent lectin staining was performed as previously described (20). Quantification of UEA-fluorescein-stained HPS was performed as previously described (20).

### Microscopy.

Light microscopy of filament morphology was performed using a Leica DM E light microscope with a 40× objective lens and equipped with a Leica DFC290 digital camera controlled by micromanager imaging software (50). Quantification of cell length and the percentage of filaments with attached heterocysts was performed as previously described (22).

Fluorescence microscopy was performed with a Leica DMIRE2 inverted fluorescence microscope equipped with either a 10× or 63× objective lens using the MetaMorph software (Molecular Devices) and a Yokogawa CSU-X1 spinning disk confocal unit with a QuantEM:5125C camera. Excitation and emission were as follows: 405 nm excitation (CUBE 405-nm, 100-mW laser at 100%; Coherent) and 460 (±25) nm emission for immunofluorescence of PilA using CF-405m, 491 nm excitation (Calypso 491-nm, 50-mW laser at 100%; Cobolt) and 525 (±25) nm emission for UEA-fluorescein-labeled HPS, and 561 nm excitation (Sapphire 561-nm 50-mW laser at 100%; Coherent) and 605 (±25.5) nm emission for cellular autofluorescence.

### Data availability.

RNA-seq data were deposited in the NCBI GEO database (GSE124969).
