# Supplementary figures and images for: A Tripartite, Hierarchical Sigma Factor Cascade Promotes Hormogonium Development in the Filamentous Cyanobacterium Nostoc punctiforme
Source: mSphere. 2019 May 1;4(3):e00231-19. doi: 10.1128/mSphere.00231-19 (PMC6495340; doi:10.1128/mSphere.00231-19)

**A**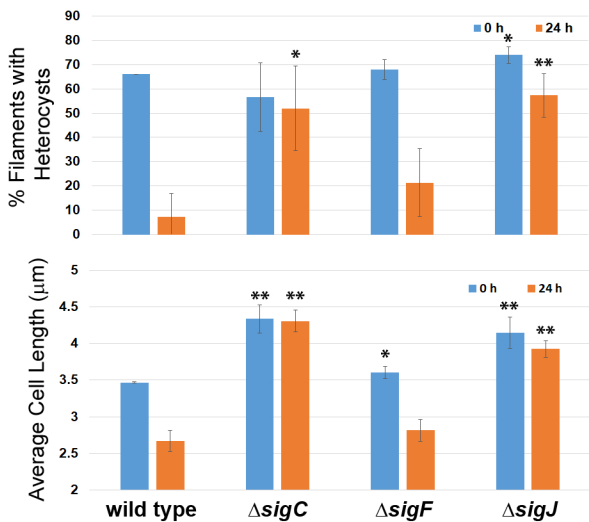**B**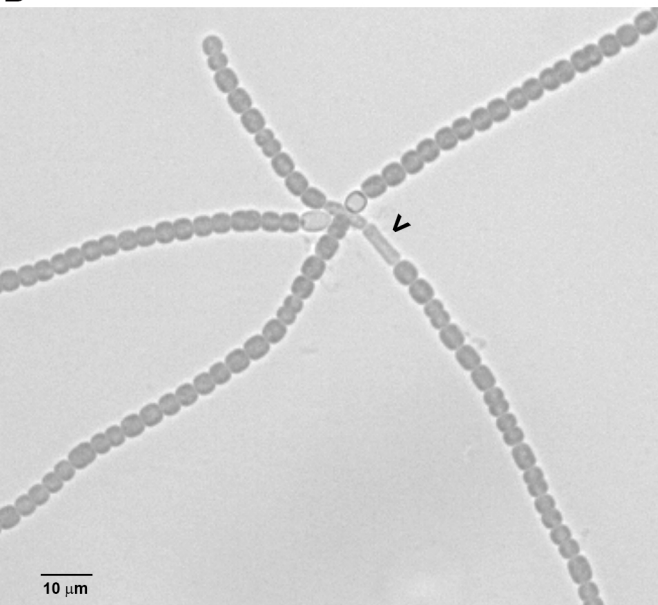

Supplement: FIG S1 [file mSphere.00231-19-sf001.pdf]

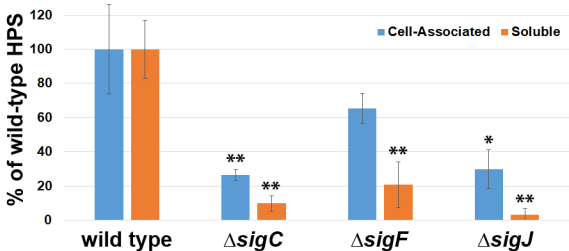

Supplement: FIG S2 [file mSphere.00231-19-sf002.pdf]

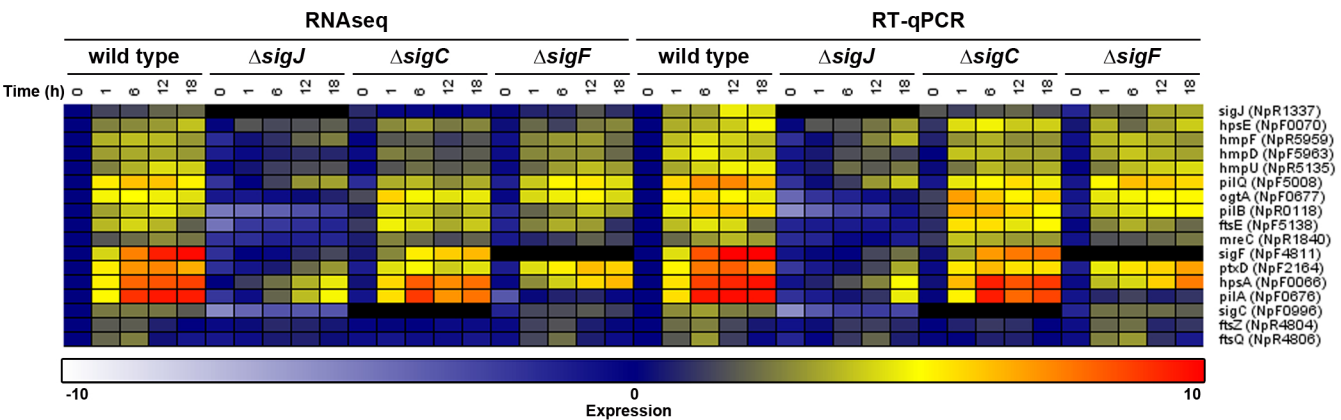

Supplement: FIG S3 [file mSphere.00231-19-sf003.pdf]
