# Supplementary material for: A Tripartite, Hierarchical Sigma Factor Cascade Promotes Hormogonium Development in the Filamentous Cyanobacterium Nostoc punctiforme
Source: mSphere. 2019 May 1;4(3):e00231-19. doi: 10.1128/mSphere.00231-19 (PMC6495340; doi:10.1128/mSphere.00231-19)
Supplement: TABLE S1 [file mSphere.00231-19-st001.pdf]

Table S1A. Strains and plasmids used in this study

Strains	Relevant Characteristic(s)	source
Nostoc punctiforme strains		
ATCC 29133	wild type	ATCC
UCD153	Laboratory derivative of *N. punctiforme* ATCC 29133 with reduced motility	(1)
TNM14139	UCD153 with Tn5-1063 insertion after nucleotide 677 of *sigC* (Npun_F0996) *	This study
TNM14211	UCD153 with Tn5-1063 insertion after nucleotide 25 of *sigC*	This study
UOP131	Δ*sigC* – encodes a group 2 sigma factor	This study
UOP132	Δ*sigJ* (Npun_R1337) – encodes a group 3 sigma factor	This study
UOP141	Δ*sigF* (Npun_F4811) – encodes a group 3 sigma factor	This study
Plasmids		
pAM504	Mobilizable shuttle vector	(2)
pRL278	Mobilizable suicide vector	(3)
pRL1063a	Suicide vector carrying Tn5-1063, a Tn5 derivative transposon	(4)
pDDR411	Suicide vector for in-frame deletion of *sigC* [1-4]†	This study
pDDR412	Suicide vector for in-frame deletion of *sigJ* [5-8]	This study
pDDR422	Suicide vector for in-frame deletion of *sigF* [9-12]	This study
pDDR453	Shuttle vector containing P_{sigC}-*sigC* [13-14]	This study
pDDR454	Shuttle vector containing P_{sigJ}-*sigJ* [15-16]	This study
pDDR455	Shuttle vector containing P_{sigF}-*sigF* [17-18]	This study

* locus tag denoted in parentheses

† numbers in brackets correspond to primers used to construct plasmid. Detailed information on primers can be found in Table S2

References

1. Campbell EL, Summers ML, Christman H, Martin ME, Meeks JC. 2007. Global gene expression patterns of Nostoc punctiforme in steady-state dinitrogen-grown heterocyst-containing cultures and at single time points during the differentiation of akinetes and hormogonia. J Bacteriol 189(14): 5247-5256.
2. Wei TF, Ramasubramanian TS, Golden JW. 1994. Anabaena sp. strain PCC 7120 ntcA gene required for growth on nitrate and heterocyst development. J Bacteriol 176(15): 4473-4482.
3. Cai YP, Wolk CP. 1990. Use of a conditionally lethal gene in Anabaena sp. strain PCC 7120 to select for double recombinants and to entrap insertion sequences. J Bacteriol 172(6): 3138-3145.
4. Wolk CP, Cai Y, Panoff JM. 1991. Use of a transposon with luciferase as a reporter to identify environmentally responsive genes in a cyanobacterium. Proc Natl Acad Sci U S A 88(12): 5355-5359.

Table S1B. Oligonucleotides used in this study

Oligonucleotide	Sequence	Number	qPCR primer set target gene
NpF0996-5'-F	ATATAGGATCCCTTGTTAGCAACCTGCAATAG	1	
NpF0996-5'-R	GATTAACTAAGTGCTGGCATAAGATCGTCTC	2	
NpF0996-3'-F	CTTATGCCAGCACTTAGTTAATCAATGATCGAGAG	3	
NpF0996-3'-R	ATATAGAGCTCTATCCACCCAAGTGCTAAAG	4	
NpR1337-5'-F	ATATAGGATCCCACACCAGTAGCTAAATACC	5	
NpR1337-5'-R	GACTACGAACCTGTTGCCATATACATTCCTTTG	6	
NpR1337-3'-F	GTATATGGCAACAGGTTCGTAGTCATGCTGAG	7	
NpR1337-3'-R	ATATAGAGCTCGACAACGTGCTGTACTTTAC	8	
NpF4811-5'-F	ATATAGGATCCTACTTTCGAGAGCGAACAGC	9	
NpF4811-5'-R	GTTAATCGTCTGCGAGTAAATTAACAATCAATTACTCAC	10	
NpF4811-3'-F	GTTAATTTACTCGCAGACGATTAACTGCAAATAAAC	11	
NpF4811-3'-R	ATATAGAGCTCCCAAATTGTTCGAGAATTACAATC	12	
PNpF0996-BamHI-F	ATATAGGATCCGAGCATACCCATGCTGTAG	13	
NpF0996-SacI-R	ATATAGAGCTCTTAACTAAGAGATTCCAAATAATCG	14	
PNpR1337-BamHI-F	ATATAGGATCCTTGTTCACCCTCCTGTTTAG	15	
NpR1337-SacI-R	ATATAGAGCTCCTACGAACCAGTGGGCATTAC	16	
PNpF4811-BamHI-F	ATATAGGATCCATTCTCTAGAGTTAGATAACTGAC	17	
NpF4811-SacI-R	ATATAGAGCTCAGTTAATCGTCTGCTACACC	18	
qNpun_r018_F1	TAAGAGCGCACCAGCAGTAT	19	*rnpB*
qNpun_r018_R1	CATTGAGCGGAACTGGTAAA	20	
qNpun_F0996-F2	GGCGATCGCAACTTCTAGTC	21	*sigC*
qNpun_F0996-R2	ACTTGGGTCGGTGTCATCTC	22	
qNpun_R1337-F1	TGAGATGCTGCACTTTTTGC	23	*sigJ*
qNpun_R1337-R2	TTTTGAGCGGCTAACTTGGT	24	
qNpun_F4811-F2	TGTTTGGGAGAATTGGTTCC	25	*sigF*
qNpun_F4811-R2	ATCCCGAGATGTTCTGCAAC	26	
qNpun_R5959-F2	CAACAACTGGAGCAGCAAAA	27	*hmpF*

qNpun_R5959-R2	CTGGAAATCCGCTTGATGTT	28	
qNpun_F5963-F2	TAGTGATGCTTTGCGTCAGG	29	*hmpD*
qNpun_F5963-R2	GCTTCACCACCTTTGAGAGC	30	
qNpun_R5135-F2	AAAAGTGCAAGCCTGTTGCT	31	*hmpU*
qNpun_R5135-R2	CGGCGCAAAACTGAAATAAT	32	
qNpun_F5008-F2	GCACCAATTCCCTGACCTTA	33	*pilQ*
qNpun_F5008-R2	ATGCTGCACCACCATCATTA	34	
qNpF0677-F2	TGCGGCAAAATAAAGTAGCA	35	*ogtA*
qNpF0677-R2	TCCTCGCTGGATTAGGTGTT	36	
qNpun_R0118-F2	AATGGTGTCGGCTACAAAGG	37	*pilB*
qNpun_R0118-R2	TCGGCTTCCAAACCAGTATC	38	
qNpun_F5138-F2	CTGTTTATCACCGGGCCAAG	39	*FtsE*
qNpun_F5138-R2	ACCCTTGAGCTTGCAGTACA	40	
qNpun_R1840-F2	GGTAGTGCTTGGGTATTGCG	41	*MreC*
qNpun_R1840-R2	CGTGATGCCAGTGGTTCTTT	42	
qNpun_F2164-F2	ATGGCTCCCCTGTTAAAGCT	43	*ptxD*
qNpun_F2164-R2	GGCTGCTAACAAACCCCAAA	44	
qNpun_F0066-F2	TGTAATGCCGCAGCTACAAC	45	*hpsA*
qNpun_F0066-R2	TGATCCCCTCCTGTAGCATC	46	
qpilA-F2	TCTGGTTGCCAACAATGGTA	47	*pilA*
qpilA-R2	ACTTCAGCACTCCGATCACC	48	
qNpun_R4804-F2	GCTCTCACTGTTGGGGTAGT	49	*ftsZ*
qNpun_R4804-R2	GGTGTTTGTTCGGGGATCAC	50	
qNpun_R4806-F2	CCCAATCTTTGTGGCGGATT	51	*ftsQ*
qNpun_R4806-R2	TACCCTGATTTGCCCCACTT	52	
qNpun_F0070-F2	GGTAGCCAAATTCACCCTGA	53	*hpsE*
qNpun_F0070-R2	TTGCCTTGAACTCTCCCAGT	54	
